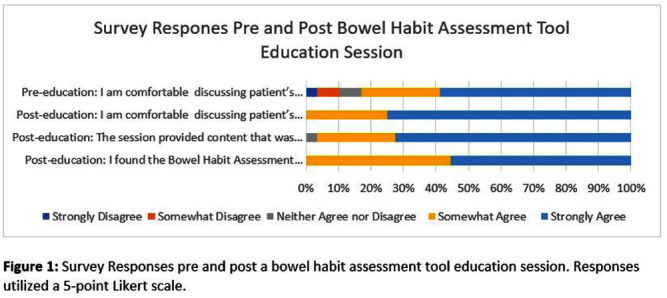# Pilot of a Bowel Habit Assessment Tool to Enable Early Identification of C. diff Infection

**DOI:** 10.1017/ash.2024.182

**Published:** 2024-09-16

**Authors:** Shelley Kon, Gunter Christine, Heidi Hough, Randi Craig, Ashley Lane, Mary Bessesen

**Affiliations:** Rocky Mountain Regional Veterans Affairs Hospital; Department of Veteran Affairs - Rocky Mountain Regional Medical Center; VA Eastern Colorado Healthcare System; University of Colorado-Denver

## Abstract

**Background:** Gastrointestinal conditions are common in hospitalized patients. Decreased mobility, dietary changes, medications and their underlying illness may alter patients’ bowel movements. It’s important for health care providers to be aware of patient’s bowel habits, especially for early identification of Clostridiodies difficile infection (CDI). Prior research has shown that patient modesty may be a barrier to discussing bowel habits with nurses and providers. This can lead to delay in diagnosis of CDI, lack of timely isolation and possible misclassification of community onset CDI cases as hospital onset (HO-CDI). **Methods:** A Bowel Habits Assessment Tool (BHAT) was developed to assist health care providers in learning skills to assess and document patient bowel habits accurately. The tool provides a structured approach to help clinicians gather relevant information, identify abnormalities, and promote effective communication with patients. The tool was developed by an infectious disease physician and modeled on existing tools utilized to take a sexual history. A team of infectious disease physicians, nurses and a gastroenterologist reviewed the tool and provided feedback. See Table 1. The tool was introduced as a pilot program at a 180 bed academically affiliated Veterans Affairs Hospital. Micro educational sessions were held to provide education about the importance of a bowel habit history, introduce the tool and teach its use in clinical care. The teaching sessions were led by an Infectious Disease physician and a nurse infection preventionist. An anonymous pre and post survey employing a 5-point Likert scale was administered to participants. All participation was voluntary. This project was reviewed and approved as a Quality Improvement by the VA Research Office, Eastern Colorado Health Care System. **Results:** Twenty nine healthcare personnel participated in the pilot. Participants included nurses (13), resident physicians (13), medical students (2) and nursing assistants (1). 59% of participants stated that they strongly agree with the statement “I am comfortable discussing patient’s bowel habits” on the pre-survey. (Question 1). This increased to 73% after the BHAT educational session. The mean difference between pre and post survey responses for question one was 0.45 (CI 0.08761 to 0.8089, p= 0.0167). All participants found the BHAT related to their work and useful, with 41% strongly agreeing and 52% somewhat agreeing that the BHAT was useful. See figure 1: Survey Responses. **Conclusions:** The effectiveness of a bowel habit assessment tool was demonstrated using a pre and post survey. BHAT improved clinicians comfort level discussing patient’s bowel habits.

**Figure:**
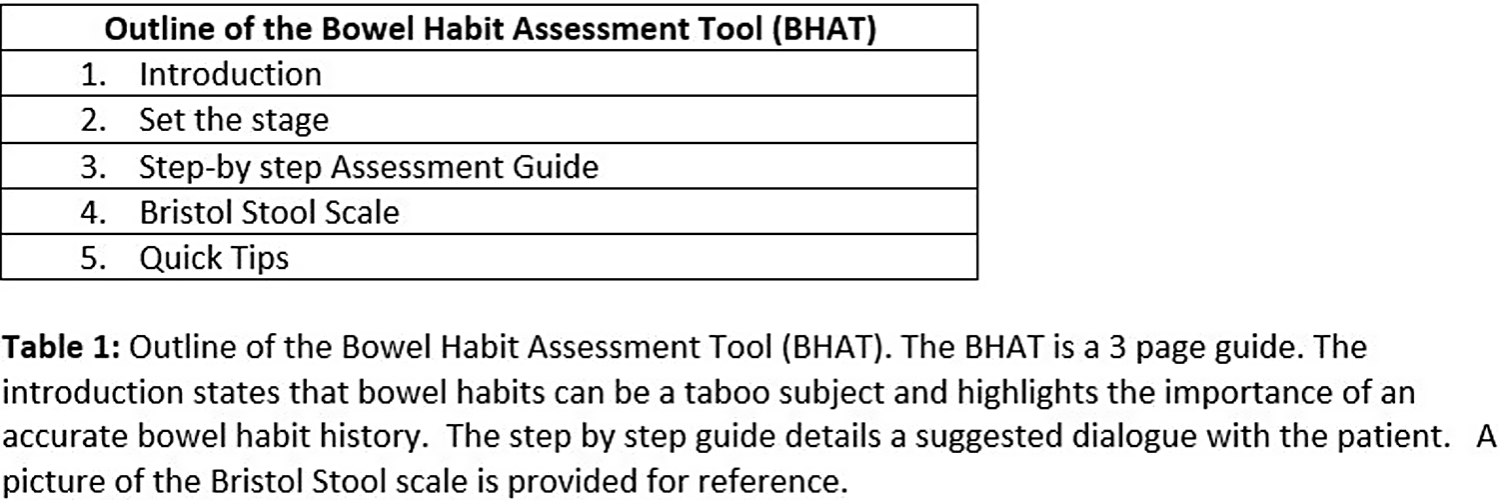

**Figure:**